# A severe case of *Streptococcal pyogenes* empyema following influenza A infection

**DOI:** 10.1186/s12890-019-0787-9

**Published:** 2019-01-28

**Authors:** Nobuhiro Asai, Hiroyuki Suematsu, Daisuke Sakanashi, Hideo Kato, Mao Hagihara, Hiroki Watanabe, Arufumi Shiota, Yusuke Koizumi, Yuka Yamagishi, Hiroshige Mikamo

**Affiliations:** 1 Department of Clinical Infectious Diseases, Aichi Medical University Hospital, 1-1 Yazakokarimata, Nagakute, 480-1195 Aichi Japan; 2 Department of Infection Control and Prevention, Aichi Medical University Hospital, 1-1 Yazakokarimata, Nagakute, 480-1195 Aichi Japan

**Keywords:** Streptococcus pyogenes, Empyema, Influenza, Co-infection

## Abstract

**Background:**

Any immunological mechanisms induced by influenza virus could cause severe secondary bacterial superinfection such as those by *Streptococcus pyogenes* [group A streptococcus (GAS)], *Streptococcus pneumoniae* or *Staphylococcus aureus.* Over recent years, the frequency of pleural empyema has increased in children with influenza infection.

We present a severe case of acute empyema caused by *S.pyogenes* after influenza A infection.

**Case presentation:**

A previously healthy 39-year old woman was diagnosed as influenza A and received oral Oseltamivir 75 mg twice daily for 5 days**.** She had no vaccination of influenza A. Although her influenza A infection improved, she complained of fever and cough to our institute. Chest radiography showed encapsulated pleural effusion of the left lung and pleural effusion which was consistent with acute empyema. Then, she was diagnosed as having acute empyema and was admitted to our institute. *Streptococcus pyogenes* was identified by pleural fluid culture on day 4. thus, MNZ was changed to clindamycin (CLDM) 600 mg three times a day. While thoracic drainage with intrapleural urokinase and combination antibiotic therapy of ceftriaxone and CLDM were performed, her general condition and chest radiographic findings were not improved. She received video-assisted thoracic debridement on day 10. After the operation, the antibiotic therapy was changed to ABPC 6 g daily iv. Due to good clinical course, the antibiotic therapy was switched to oral amoxicillin 500 mg three times daily on day 28. Then, she was discharged.

**Conclusion:**

Influenza A virus infection could lead to severe GAS infection, while the latter can occur in otherwise healthy individual as well. Physician must consider the possibility of severe GAS infection after influenza A infection.

## Background

*Streptococcus pyogenes* is a common cause of pharyngitis, deep neck abscesses, and skin and soft tissue infections [1]. It can occasionally cause toxic shock syndrome, severe pneumonia or meningitis, resulting in a high mortality of up to 20%. Also, it has been reported that *S.pyogenes* is a common bacterial pathogen of viral co-infection with varicella [2], Epstein-Barr virus [3], and influenza A virus (IAV) [4].

We present a severe case of acute empyema caused by *S.pyogenes* after influenza A infection.

## Case presentation

**Fig. 1:**
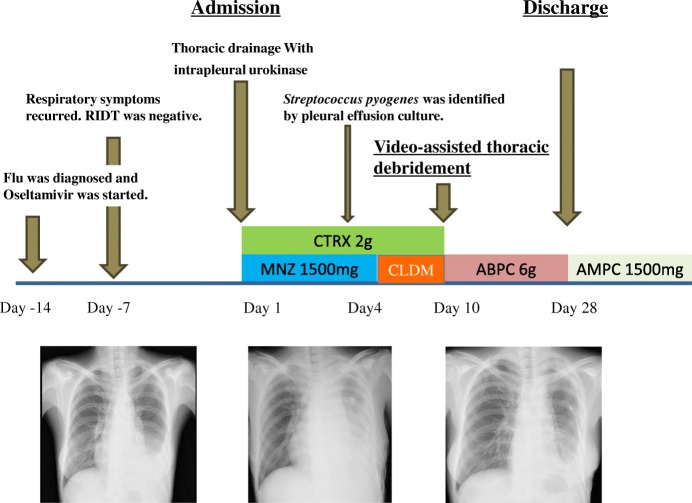
Chest X-ray shows pleural effusions in the left lung

**Fig. 2:**
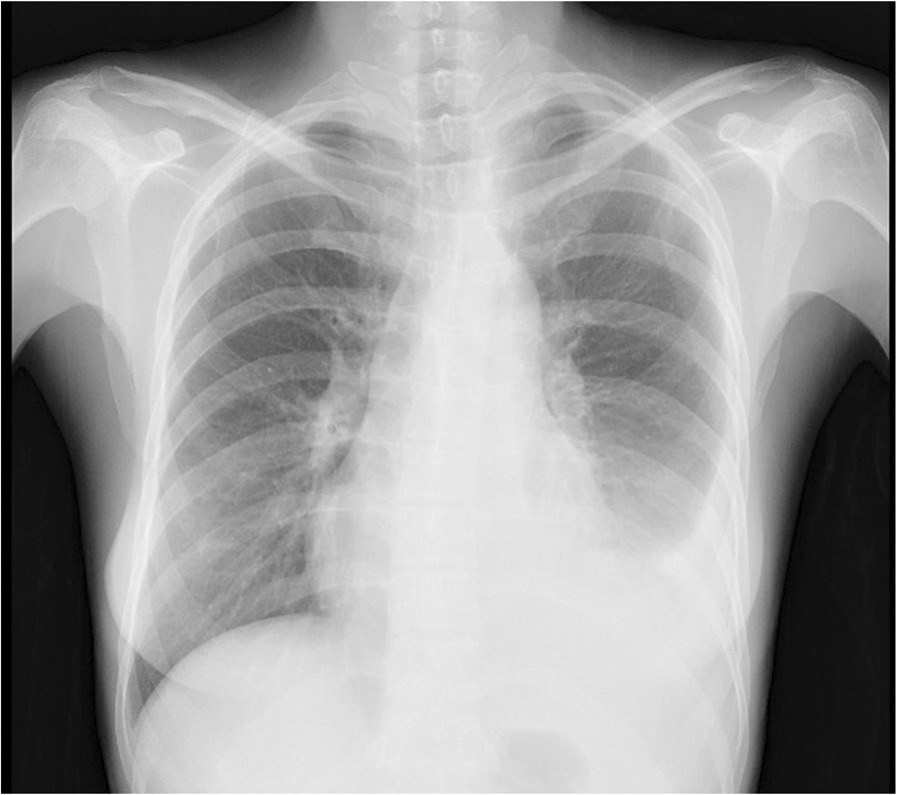
Chest CT shows pleural loculated effusions in the left lung

**Fig. 3:**
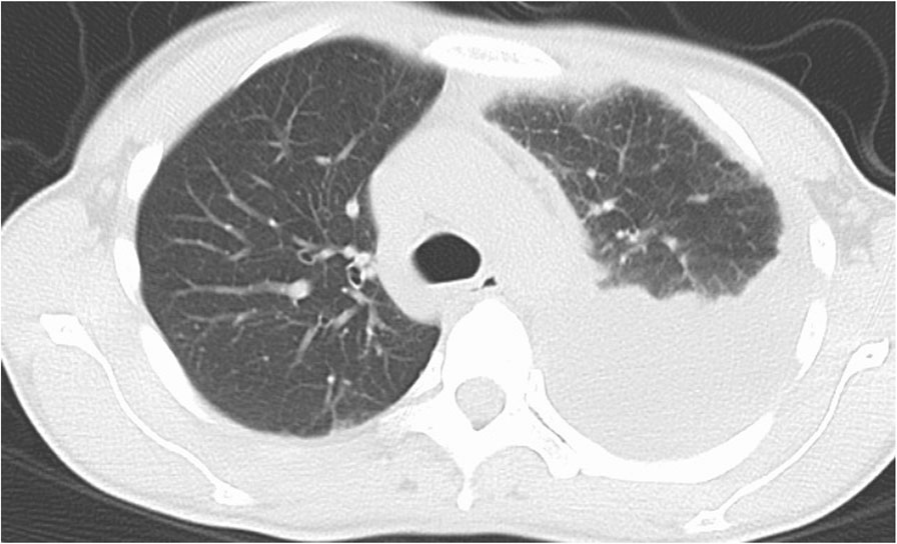
shows a clinical course in the table

A previously healthy 39-year old woman was diagnosed as having influenza A virus infection by rapid influenza diagnostic test (RIDT) in a clinic, and received oral Oseltamivir 75 mg twice daily for 5 days. The clinical course is shown in Fig. 1. While influenza like illness was improved once, fever and cough recurred on day 7 after the onset of flu. At this time, RIDT was performed, showing that the result was negative at the clinic. She complained of fever, cough and the left chest pain and presented to our institute on day 14 after the onset of the flu. RIDT was performed and the result was again negative. The data representing the inflammatory reactions were elevated (Table 1) and the chest radiography showed encapsulated pleural effusion of the left lung (Figs 2 and 3). Pleural fluid from the initial thoracentesis was pus, and showed an increase in cell counts with neutrophil predominance. Thus, she was diagnosed as having acute empyema. Thoracic drainage with intrapleural urokinase and antibiotic therapy of ceftriaxone (CTRX) 2 g and metronidazole (MNZ) were started. Pleural fluid cultures from the initial thoracentesis grew *Streptococcus pyogenes* on day 4. Thus, MNZ was changed to clindamycin (CLDM) 600 mg three times a day. On day 10 after the antibiotic therapy with thoracic drainage was started, she received video-assisted thoracic debridement due to worsening of the patients’ general condition and infiltrations by chest radiography. After the operation, the patient’s condition improved and antibiotic de-escalation was performed to ampicillin 6 g daily iv. Due to patient’ good condition, antibiotic therapy was switched to oral amoxicillin 500 mg three times daily on day 28. Then, she was discharged. During this six months, recurrence of the infection was not observed.

**Table 1:** Results of laboratory examinations on admission

Blood count (normal range)	
White blood cell count (5000–8000)	22,200 /μl
Neutrophil (%) (40–60)	91.0
Eosinophil (%) (0-4.5)	0
Basophil (%) (0-1.9)	0
Monocyte (%) (3.8–5.5)	6.0
Lymphocyte; (%) (30.3-40.5)	3.0
Red blood cell count (370–480 × 10^4^)	462 × 10^4^ /μl
Hemoglobin (11.4–14.8)	13.9 g/dl
Platelet count (18.0–35.0)	32.7 × 10^4^ /μl
Blood chemistry (normal range)	
Total protein (6.7–8.3)	7.2 g/dl
Albumin (4.0–5.0)	2.0 g/dl
Total bilirubin (0.3–1.2)	0.78 mg/dl
Aspartate amino transferase (13–33)	25 IU/l
Alanine amino transferase (6–27)	14 IU/l
Lactate dehydrogenase (119–229)	269 IU/l
Alkaline phosphatase (115–359)	315 IU/l
γ-Glutamyl transpeptidase (10–47)	18 IU/l
Blood urea nitrogen (8–22)	7.8 mg/dl
Creatinine (0.4–0.7)	0.66 mg/dl
Sodium (138–146)	130 mEq/l
Potassium (3.6–4.9)	3.5 mEq/l
Chloride (99–109)	95 mEq/l
Glucose (70–109)	152 mg/dl
Amylase (37–125)	20 U/l
Serum	
C-reactive protein (≦0.03)	32.2 mg/dl

## Discussion

The group A streptococcus (GAS) can colonize humans asymptomatically or often causes infections of the pharynx and the skin. It could cause invasive infections including bacteremia, pneumonia, and toxic shock syndrome (TSS), resulting in the high mortality of up to 20% [1].

Over recent years, the frequency of pleural empyema has increased in children with influenza A infection [5–7]. This tendency could be explained by influenza virus induced-immunological mechanism. Barthelemy documented that influenza A virus-induced release of interleukin (IL)-10 inhibited the antimicrobial activities of invariant natural killer T cells during invasive pneumococcal superinfection [8]. Robinson reported that influenza A virus inhibits bacterial-induced IL-1β production and impairs host defense against bacterial infection [9]. These cytokines by influenza virus and regulatory T cell could play an important role in the immunological mechanisms underlying influenza virus-GAS superinfection. Some reported that secondary bacterial superinfection could occur in one week after influenza infection [10, 11]. In our case, respiratory symptoms surfaced in 7 days after influenza A infection as previous reports. It is consistent with clinical course of secondary bacterial superinfection after influenza infection.

Previous report documented that vaccination of influenza A virus could prevent secondary GAS infection. In one study, mice were vaccinated against influenza virus and subsequently infected with an H1N1 virus 2 days prior to infection with GAS [12, 13]. The mortality of mice vaccinated and unvaccinated with IAV were 15 and 78%, respectively. Similarly, during the observation period of 1993–2001, the incidence of *S.pyogenes*-varicella coinfections reduced from 27% of pre-varicella vaccination era to only 2% after the 1995 when the varicella vaccine became available [14].

These results suggest that vaccination of IAV could prevent secondary GAS infection. Our patient had not received IAV vaccination, and thus, severe GAS infection could occur after influenza A infection. Vaccination of IAV is important not only for preventing influenza infection, but also for secondary infections after flu such as pneumococcal, or GAS infection.

As for treatments, the patient empirically received combination therapy of CTRX and MNZ. *S. pyogenes* has the cell wall M-protein which inhibits complement activation and decreases phagocytosis. Furthermore, *S.pyogenes* produce exotoxins that are capable of direct T-cell activation without antigen processing by antigen-processing cells, resulting in cytokine storm. Clindamycin (CLDM) would be effective to deactivate M-protein and these exotoxins, resulting in a favorable outcome [15, 16]. Some documented that efficacy and rationale for use of intravenous immunoglobulin therapy (IVIG) in streptococcal TSS [17, 18]. It has been reported that several mechanisms were thought to be opsonization of GAS for phagocytic killing, neutralization of streptococcal toxins, inhibition of T cell proliferation, and inhibition of inflammatory cytokines such as TNF-alpha and IL-6 [19, 20]. While its efficacy has been reported, IVIG is not standardized evidence-based in the treatment for invasive GAS infections.

In this case, combination therapy with CLDM as initial treatment or IVIG should have been considered for prevention of exacerbation of patient’s condition.

In conclusion, influenza A virus infection could lead to severe GAS infection, while the latter can occur in otherwise healthy individual as well. Physician must consider the possibility of severe GAS infection after influenza A infection. Also, vaccination of IAV could prevent secondary severe GAS infection.

## Abbreviations

CLDM: Clindamycin
CTRX: Ceftriaxone
GAS: Group A streptococcus
IAV: Influenza A virus
IL: Interleukin
IVIG: Intravenous immunoglobulin therapy
MNZ: Metronidazole
RIDT: Rapid influenza diagnostic test
TSS: Toxic shock syndrome
